# Moral disengagement and willingness to behave unethically against ex-partner in a child custody dispute

**DOI:** 10.1371/journal.pone.0213662

**Published:** 2019-03-13

**Authors:** Miguel Clemente, Pablo Espinosa, Dolores Padilla

**Affiliations:** Departamento de Psicología, Universidade da Coruña, La Coruña, Spain; Western Sydney University, AUSTRALIA

## Abstract

The current study examines the role of moral disengagement on the likelihood of making false allegations or retaliating against the partner in a child custody dispute. Moral disengagement strategies can be useful to explain this tendency to harm their partner in a custody dispute, because they help reduce the aversive state caused by the dissonance provoked when the ethical principles of the individual do not match their behavior. An individual that is able to lessen the negative affect anticipated before committing a transgression, would be more likely to engage in it. A sample of 1097 Spanish adults who had experienced a break up with their partner and had children participated in the study. They were evenly divided by sex and their mean age was 39.95 (*SD* = 8.89). They answered to a vignette depicting a child custody dispute during a break up process and answered to a series of questions regarding whether they would be willing to make false allegations or to take retaliatory action against their former partner. They also answered to questionnaires on moral disengagement and the “dark triad” of personality. Results show that moral disengagement is a significant predictor of false allegations and retaliatory action, stronger than any of the variables included in the “dark triad”, and predicts willingness to harm the partner beyond the common core of dark traits. We did not find gender differences in inclination to harm the partner, although men are more prone to the use of moral disengagement strategies than women. Still, we found that the type of moral disengagement that better predicts these tendencies is different for men and women. Men significantly favored reconstrual strategies that include moral justification, advantageous comparison and euphemistic labelling, while the best predictor for women are strategies focused on the recipient, like attribution of blame or dehumanization.

## Introduction

To date, no research has been conducted on the role of moral disengagement in a court setting or in a child custody dispute. This study is aimed at examining the role of moral disengagement in individuals involved in a hypothetical separation process involving an argument over the custody of the couple’s children and the potential differences between men and women. Ex-partners in this context may feel inclined to harm their former spouse and the use of moral disengagement strategies may facilitate this behavior.

It is not clear how many people are willing to make false allegations or otherwise behave unethically in a court setting. For instance, regarding intimate partner violence there has been controversy for a long time about the rate of false and further research is needed to clarify the issue [1]. Only a few studies offer an estimate of the number of false allegations. Trocme and Bala [2] estimated that in Canada, 4% of child maltreatment cases, allegations were intentionally false, and that a further 31% were unsubstantiated (false, but in good faith or not intentional). While under no custody dispute the intentionally false and unsubstantiated cases were 3% and 31% respectively, under a custody dispute intentionally false cases rose to 12% and unsubstantiated cases to 34%. Comparing custodial (usually the mother) and non-custodial parents, the study found that 15% of reports by the non-custodial parent were false and 37% unsubstantiated. In the case of the custodial parent 2% of reports were false and 27% unsubstantiated. So while these figures show that making false allegations is not commonplace, the rates are higher in cases of parental separation than in other contexts, particularly for the non-custodial parent. Specifically, in their reviews of research on child sexual abuse during divorce, Penfold [3] states that 8% to 16.5% of reports are false and Smit, Antokolskaia and Bijleveld [4], found that one out seven cases are unfounded. In a different but related domain, the General Council of the Judiciary in Spain [5] found very low numbers (0.4%) of false reports in intimate partner violence cases. So, although the rate of unethical behavior is low, it warrants further examination of the variables involved in the process.

Moral disengagement is a key variable in explaining unethical behavior. According to Bandura’s model of moral disengagement [6,7], when people commit a transgression that violates their personal standards, they tend to experience negative affect coming from the cognitive dissonance between their behavior and their principles. However, they often find ways to minimize this negative affect by using a series of cognitive strategies to reduce the effect of behaving against one’s own standards and to disengage from the moral sanctions of such behavior. Furthermore, this disengagement may happen after committing the transgression or anticipatorily. If people anticipate ways to disengage from the moral self-sanctions of their negative behavior, they will feel less guilty and will be more likely commit the transgression. Additionally, people who behave negatively, tend to use more moral disengagement strategies to avoid self-sanctions. Thus, moral disengagement mechanisms are a form of self-serving bias that allow us to behave against our own principles without feeling bad about it and they help restructuring antisocial behaviors into bening ones. People who are high moral disengagers feel less guilty about transgressions [8]. Individuals use moral disengagement strategies as a mechanism of moral rationalization to convince themselves that their behavior does not violate their moral standards, whether it is a trifle misconduct or a serious crime.

Bandura has described three types of mechanisms of moral disengagement: The first type of mechanisms focus on the transgression itself, and aim at redefining the behavior in a more positive way and providing self-approval for transgressions. According to Bandura *et al*. [6] these reconstrual strategies include the mechanisms of moral justification (the transgression is personally as personally or socially acceptable and serving a higher purpose); advantageous comparison (the behavior is seen in a more positive light compared to worse transgressions) and euphemistic language (the behavior is given a more benign label so that it sounds less negative). Another type of mechanisms focuses on the role that the individual has played in the transgression or his/her personal agency, or tries to dissociate detrimental actions from their consequences. Personal agency allows the individual to refuse acknowledgement of the harmful outcomes of his or her behavior. It includes the strategies of displacement of responsibility (responsibility is placed on someone else or the circumstances); diffusion of responsibility (individuals acknowledge only a small part of responsibility or a minor role in the harmful outcomes because responsibility is shared with other people who took part) and distortion of consequences (distorting or disregarding the harm caused by their behavior or refusing to acknowledge it). The final set of strategies focuses on the recipient of the transgression and includes the strategies of attribution of blame (blaming the victim or claiming that the recipient provoked the transgression with his or her behavior); or dehumanization (degrading and attributing to the recipient negative qualities that make the victim less than human and undeserving of compassion or consideration).

Among other variables studied in relation to transgressions, there is an abundance of research that focuses on a set of three variables: Machiavellianism, Narcissism and psychopathy, defined as the “dark triad” of personality. Jointly or individually these variables have been often proposed as predictors of unethical behaviors [9]. The “dark triad” has been associated specifically to intimate partner violence [10]). Moral disengagement is significantly related to a number of variables associated positively or negatively to the dark triad and transgressions. Detert, Treviño, and Sweitzer [11] found that moral disengagement is positively correlated to trait cynicism and chance locus of control (which leads to not feeling responsible for one’s outcomes) and negatively related to moral identity and empathy. Additionally, it operates as a mediator for the relationship between these variables and unethical behavior. Since empathy is the tendency to be concerned with the feelings and needs of others, and moral disengagement cares about self-serving motives, both dispositions conflict. An individual high in empathy would have a hard time justifying or disregarding negative consequences for others or dehumanizing the targets of their detrimental behaviors, and may in fact inhibit moral disengagement.

Moore, Detert, Treviño, Baker and Mayer [12] also found that moral disengagement significantly relates to unethical behavior, moral identity, empathy and Machiavellianism. Similarly, Egan, Hughes and Palmer [13] found that moral disengagement is related to all variables in the dark triad: Machiavellianism, Narcissism and psychopathy. Furthermore, they found that it is a better predictor of unethical attitudes than the dark triad.

Furthermore, Moshagen, Hillbig and Zettler [14] state that most previous research on dark traits has usually been focused on individual traits or small subsets of them, so it is unclear if a specific trait has a unique role in the explanation of behavior or it rather measures a general dispositional tendency. They argue that there is general dispositional tendency that encompasses both moral disengagement and the “dark triad” traits. This broader tendency, namely the Dark Factor of Personality, would not only explain the underlying tendencies common to Moral disengagement, Machiavellianism, Narcissism and Psychopathy, but also other traits like Egoism, Psychological entitlement, Sadism, Self-interest and Spitefulness. All of these traits would be specific expressions of a common core tendency of disregarding others’ outcomes in favor of self-interest or a behavioral bias towards the self [15]. However, we believe that moral disengagement plays a distinctive role in the explanation of unethical behavior, since it places the focus of behavior on avoiding self-sanctions associated with trespassing personal moral standards, whereas the common core of dark traits subsumed in the Dark Factor of personality is also concerned with beliefs that serve as justifications for negative behavior, but from a broader perspective (e.g. cinycism, maximizing personal outcomes despite others or a sense of entitlement or superiority), that comprises justifications for the adequacy or benefits of behaviors that deviate from social or moral norms [14]. In contrast with this common core, moral disengagement focuses specifically on justifying behavior that deviates from personal moral standards so as to avoid the aversive consequences of violating them.

Individuals that disengage from self-sanctions act in self-interest, but they need to rationalize immoral behavior into being moral and cannot simply ignore moral principles when they compete with another desired outcome. Behaviors that are inconsistent with one’s own moral principles result in an aversive state of cognitive dissonance that the individual is motivated to avoid. When individuals are motivated towards antisocial behavior their cognitions become distorted in order to build a rationalization that complies with their desired outcome and their personal moral principles. This rationalization works both to convince themselves and to convince others that the behavior is acceptable [16].

Another concern is the difference between genders, both in moral disengagement and in the tendency to harm the partner in a custody dispute. Men are generally found to score higher in moral disengagement [6,7], but are not more inclined to act against their partner or children in related settings [3,17]. Although moral disengagement is positively related to unethical behaviors, gender and other variables play a moderating role, so that gender differences in moral disengagement do not always translate into behavior differences. For instance, Samnani, Salamon and Singh [18] found that men high in moral disengagement showed more unethical behavior than women high in moral disengagement, only when comparing groups with high negative affect. Also, women are more overall more empathetic than men, and gender differences in dark traits are mediated by empathy [17]. Arguably, the relationship between gender, antisocial behavior, moral disengagement or other dark traits is also mediated by empathy. The current study specific goals are examining the relationship between moral disengagement and inclination to harm the partner in child custody disputes; checking whether moral disengagement is a better predictor of unethical attitudes in this setting compared to variables in the dark triad and a general Dark Factor of personality, and examining gender differences in attitudes towards unethical behaviors against their partner and in the use of moral disengagement mechanisms.

Since moral disengagement is linked to unethical behavior in other domains, we expect the same for unethical behavior in custody disputes, and thus our first hypothesis is:

*H1*: *Moral disengagement would be a significant predictor of willingness to harm the partner in a child custody dispute*.

Moore *et al*. [12] found stronger correlations of self-reported transgressions with moral disengagement than with Machiavellianism. As they argue, moral disengagement may be a stronger predictor of unethical behavior than other variables in the dark triad, like Machiavellianism, because it focuses on the individual’s general tendency to disengage from self-sanctions that would prevent transgressions, while Machiavellianism taps on more specific behaviors. So, our second hypothesis is:

*H2*: *Moral disengagement would be a significantly stronger predictor of willingness to harm the partner in a child custody dispute compared to variables in the dark triad*.

Additionally, moral disengagement shares with other dark traits a common tendency related to detrimental behavior, captured by the Dark Factor of Personality [14]. Nevertheless, as stated above, we believe that it is partly distinct from this common tendency. In this sense, we propose a third hypothesis:

*H3*: *Moral disengagement would still be a predictor of* willingness to harm the partner *in a child custody dispute when controlling for the Dark Factor of Personality*.

Our fourth hypothesis regards behavioral differences between men and women. Hamel Desmarais, Nicholls, Malley, Morrison and Aaronson, [19] state that the rates of partner aggression are similar for males and females. Additionally, Penfold, [3] states that men and women are equally likely to make false accusations of child sexual abuse. We expect these relationships to translate to unethical or harmful behaviors in a court setting.

*H4*: *Attitudes toward unethical behavior in a child custody dispute would not be significantly different in men and women*.

Lastly, we expect to find that men show higher levels of moral disengagement than females, and we will also examine whether men and women use the same type of moral disengagement strategies in relation to willingness to harm the partner in a child custody dispute.

*H5*: *Men will significantly use more moral disengagement strategies than women*.
*H6*: *There will be differences in the strategies that men and women use to morally disengage from unethical attitudes related to child custody disputes*.

## Method

### Participants

Participants in this study were 1097 Spanish adults who had been involved in a separation process. They were evenly divided by sex (50% men and 50% women) and their mean age was 39.95 years (*SD* = 8.89). All of them were parents of underage children that had experienced a couple break-up. This feature was important for our study, since they had experienced issues related to the scenarios described later and would be better able to comprehend the nuances in them compared to other participants. They were also more representative of the population exposed to custody disputes. The study has the approval of the Social Psychology area at the Department of Psychology at Universidade da Coruña and participants gave their informed consent to participate in the study and for the publication of anonymous raw data.

### Procedure

The sample was incidental and to collect the data we were aided by a team of university undergraduates who received course credit in return. Participants were contacted by the surveyors asked to complete an online survey. Prior to answering the measures in the study, they gave their informed consent and were debriefed after completing the questionnaires.

### Measures

Participants responded to a set of questionnaires to measure moral disengagement, Machiavellianism, Narcissism, and psychopathy, and to a scenario depicting a break up with their partner without agreement on child custody. The scenario included a questionnaire that asked participants whether they would be willing to lie in court and to retaliate against their partner after he/she files a complaint in court against the participant.

#### Moral disengagement

To measure moral disengagement we used the *Propensity to Morally Disengage* scale (PMD) by Moore *et al*. [12] which was designed for adults in any type of context. It is comprised of 24 items in a 7-point Likert scale ranging from *strongly disagree* to *strongly agree*. These items measure each of the eight moral disengagement strategies proposed by Bandura [6]. A sample item is "It is alright to lie to keep your friends out of trouble." (Measuring moral justification mechanisms). Additionally, it has a low correlation with social desirability measures so it is not prone to be contaminated by this bias.

#### Machiavellianism

To measure participant’s Machiavellianism, we selected the Mach-IV Scale (NPI) [20]. This scale is the widely used in research and has adequate psychometric properties. It is composed of 20 items on a 7-point Likert format ranging from 7 (high Machiavellianism) to 1 (low Machiavellianism) and 10 of the items are reversely scored to prevent response bias. It’s validity has been tested in a Spanish sample [21].

#### Narcissism

We used the Spanish version of the Narcissistic Personality Inventory [22]. It includes 40 items on a 6-point Likert scale, from “*strongly disagree*” to “*strongly agree*”.

#### Psychopathy

As a measure of psychopathy, we asked participants to complete Levenson’s Primary and Secondary Psychopathy Scales (LPSP) [23]. This scale is composed of 26 items. The first 16 measure primary psychopathy, and the last 10 measure secondary psychopathy. The response form is a 5-point Likert-type ranging from “*strongly disagree*” to “*strongly agree*”.

#### Custody scenarios

We developed a vignette in which we asked participants to imagine themselves in a break up situation with their partner in which there is controversy over the custody of their two children. In this scenario, an expert advises the respondent to make some false statements in court to improve his/her chances of success. Then the participant had to answer whether they would issue 8 different statements (e.g. “My partner treated my children badly, despised and insulted them”). They were asked to use a 4-point scale with the following options: “I would never do it” (1); “I don’t think I would do it” (2); “I might do it” (3) and “I would surely do it” (4). Next, they were asked to imagine that two years after the break up there still is conflict over custody of the children and their partner has filed a complaint in court against them. Then they had to respond on the same scale whether they would carry out 7 different retaliatory actions (e.g. “Stop compulsory alimony payments for the children”).

### Data analysis

First, we checked the reliability of the measures used in the study and the correlations between the variables analyzed. A series of *z*-tests were performed to check for significant differences between correlations.

In order to test whether moral disengagement measures different aspects from a Dark common general factor, a bifactor approach is useful [14,24,25]. In a bifactor model, each indicator is specified to load on a first-order general factor and also on a first-order specific factor, so as to capture the common variance shared by all items and the variance explained by the specific factors exclusively. So, specific factors account for the remaining variance not included in the general factor.

Although, we have not tested many of the dark traits that comprise the Dark Factor of Personality, it is a robust construct independent of any particular trait and its predictive capability remains even after removing relevant indicators [14]. So to test the specific role of moral disengagement within the larger construct of the Dark factor, we followed two routes. First we calculated the partial correlation of moral disengagement and willingness to harm the partner in a child custody dispute controlling for the weighted mean of all the indicators of dark traits in the study in order to represent the Dark factor. We also ran a SEM bifactor model using Amos 23.0.0, where all indicators of dark traits loaded on a general factor, used as proxy for the Dark Factor of Personality and also on individual factors representing moral disengagement, psychopathy, Machiavellianism and Narcissism. All these factors were set as predictors of a general factor of willingness to harm the partner behavior in a custody dispute, comprised of all the variables used in the vignettes. Since Chi-square is influenced by sample size [26] and model size [27], both of which are large in our study, we relied on other goodness of fit statistics, like SRMR and RMSEA.

*T*-tests were also run to examine sex differences. Additionally, regression analyses were carried out for men and women to check which types of moral disengagement strategies predicted lying in court and retaliating against the partner and *z*-scores were obtained to check significant differences between variables in the regression analyses for men and women.

## Results

### Reliability analyses

The measure of moral disengagement used showed a much higher reliability (α = .95) than each of the measures belonging to the dark triad. Machiavellianism (α = .63), Narcissism (α = .78), psychoticism (α = .77) showed less consistent results.

The scale regarding willingness to lie in court showed a high reliability (α = .93) and so did the scale for retaliating against the partner (α = .93).

### Correlational analyses

Moral disengagement was found to be related to both unethical behavior and variables in the dark triad. It showed a significant correlation to Machiavellianism (*r* = .53, *p* < .001), Narcissism (*r* = .23, *p* < .001) and psychopathy (*r* = .53, *p* < .001). As it turns out, moral disengagement is a strong predictor of willingness to harm the partner, even surpassing variables in the dark triad construct, as Table 1 shows.

**Table 1:** Zero-order correlations between unethical judicial attitudes, global moral disengagement score, dark triad variables and D-score.

	Lying in court	Retaliating against partner
Moral disengagement	.33*	.37*
Machiavellianism	.20*	.23*
Narcissism	.16*	.11*
Psychopathy	.17*	.22*
Dark Factor score	.32*	.35*

*N* = 1097.

*p < .01.

Following the directions by Steiger [28], we compared correlations with moral disengagement to correlations with variables in the dark triad and found significant differences in every case. Moral disengagement showed significantly higher correlations with lying in court than Machiavellianism (*z* = 3.28, *p* < .01), Narcissism (*z* = 4.24, *p* < .01) and Psychopathy (*z* = 4.00, *p* < .01). Moral disengagement also showed significantly higher correlations with retaliating against partner than Machiavellianism (*z* = 3.61, *p* < .01), Narcissism (*z* = 6.50, *p* < .01) and Psychopathy (*z* = 3.85, *p* < .01). We also compared correlations with moral disengagement to correlations with the Dark Factor score, but differences were non-significant.

### Differences with the dark factor of personality

The partial correlations, controlling for the Dark factor estimate, of moral disengagement with lying in court (*r* = .11, *p* < .01) and retaliating against partner (*r* = .14, *p* < .01) were significant. These correlations were also significantly different from the *zero-order* correlations of these variables (*z* = 5.43, *p* < .01) and (*z* = 5.79, *p* < .01), showing that although moral disengagement plays an unique role in explaining the target attitudes, there is also an underlying factor that accounts for significant differences.

The bifactor model to test whether moral disengagement had a the distinctive role in the explanation of the willingness to harm the partner in a custody dispute or would rather be subsumed into a more general Dark factor, showed that, although chi-square was significant (χ^2^(9595) = 27713.6, *p* < .001), other fit indices showed and acceptable fit (*SRMR* = .055; *RMSEA* = .042 (90% CI: .041-.042).

The general Dark factor of personality significantly predicted willingness to harm the partner in a custody dispute (*β* = .36, *p* < .001). Specific moral disengagement was also a significant distinctive predictor (*β* = -.18, *p* < .001) (Note that the loadings in the moral disengagement factor for moral disengagement indicators were all negative, so in fact the factor measured rejection of moral disengagement, so the regression *β* is also negative). The only other significant factor was Narcissism (*β* = .10, *p* < .05), indicating that the Dark Factor accounted for the variance of Psychoticism and Machiavellianism.

### Gender differences

To compare differences between men and women in the analyzed variables, we ran a series of *t*-tests. We found no significant differences between men and women for lying on court and for retaliating against the partner and the overall mean score for participants was rather low (*M* = 1.63, *SD* = .66 for lying in court and *M* = 1.40, *SD* = .63 for retaliating against partner, on a 4-point scale). Overall the rates for lying in court and retaliating against partner where rather low. Only 3.9% of the total sample scored 3 or higher in the scale.

The comparison between genders as regards variables in the dark triad showed only significant differences for Machiavellianism, with men scoring higher than women (*t*(1095) = 5.35, *p* < .001, men *M* = 2.91, *SD* = .39; women *M* = 2.78, *SD* = .41). The tests yielded significant results for moral disengagement, with men showing higher scores than women (*t*(1095) = 5.44, *p* < .001, men *M* = 3.27, *SD* = 1.09; women *M* = 2.90, *SD* = 1.14). We checked whether this pattern also appeared for each of the three moral disengagement strategies, and found that for every type, men showed more tendencies to morally disengage than women. Thus, men scored significantly higher in reconstrual strategies (*t*(1095) = 6.57, *p* < .001, men *M* = 3.40, *SD* = 1.14; women *M* = 2.94, *SD* = 1.19), personal agency strategies *t*(1095) = 4.30, *p* < .001, men *M* = 3.19, *SD* = 1.17; women *M* = 2.88, *SD* = 1.23) and recipient-based strategies(*t*(1095) = 4.25, *p* < .001, men *M* = 3.19, *SD* = 1.15; women *M* = 2.88, *SD* = 1.20). Individually, as Table 2 shows, all moral disengagement strategies correlated significantly with both lying in court and retaliating against the partner.

**Table 2:** Zero-order correlations between unethical judicial attitudes and moral disengagement strategies.

		Reconstrual	Personal agency	Recipient
Lying in court	Men	.36*	.30*	.28*
	Women	.29*	.29*	.31*
Retaliating against partner	Men	.37*	.35*	.31*
	Women	.32*	.36*	.38*

Men (*N* = 511); Women (*N* = 586).

*p < .01.

But in order to check whether there are differences in the strategies that men and women choose in relation to lying in court or retaliating against the partner, we ran regression analyses entering the three types of disengagement strategies simultaneously as predictors of both lying in court and retaliating against the partner.

As regards lying in court, moral disengagement strategies contributed significantly to the explanation of the variance for both men (*R*^2^ = .13, *F*(3,510) = 25.82, *p* < .001) and women (*R*^2^ = .11, *F*(3,585) = 24.72, *p* < .001). Controlling for the other strategies, reconstrual strategies was the only significant predictor for men (*β* = .23, *t*(510) = 4.73, *p* < .001), and recipient strategies the only significant predictor for women (*β* = .15, *t*(585) = 3.90, *p* < .001).

As for retaliating against the partner, we found a very similar pattern. Moral disengagement strategies explained a significant proportion of the variance for both men (*R*^2^ = .14, *F*(3,510) = 28.25, *p* < .001) and women (*R*^2^ = .15, *F*(3,585) = 34.41, *p* < .001). We found that reconstrual strategies was the only significant predictor for men (*β* = .15, *t*(510) = 3.36, *p* < .001). For women, both recipient (*β* = .14, *t*(585) = 3.97, *p* < .001) and personal agency (*β* = .10, *t*(585) = 2.16, *p* < .001) strategies were significant.

Next, we checked whether moral disengagement strategies significantly differed between men and women as predictors for unethical attitudes against the partner. Following the procedure recommended by Paternoster, Brame, Mazerolle and Piquero [29] we checked for significant gender differences the regression coefficients. Hence, we obtained the *z* scores for the differences between betas for men and women for each of the moral disengagement strategies as predictors of unethical attitudes, when controlling for each other. When predicting lying in court we obtained a *z*-score of 3.09 (*p* < .01) for gender differences in reconstrual strategies, and a *z*-score of -2.80 (*p* < .01) for gender differences in recipient strategies. We found no significant gender differences for personal agency strategies as a predictor of lying in court. A similar pattern was obtained when predicting retaliation against the partner. Men and women differed significantly in the use of reconstrual strategies (*z* = 2.99, *p* < .01) and in the use of recipient-based strategies (*z* = 2.60, *p* < .01), but no gender differences were found in the use of personal agency strategies.

## Discussion

The current study highlights moral disengagement as a key variable in the explanation of false allegations and unethical attitudes. Our results, as predicted by our first hypothesis show the relevance of moral disengagement mechanisms to explain unethical behavioral tendencies in a specific setting like child custody disputes. In line with our second hypothesis and previous research [12], moral disengagement appears to be a significantly stronger predictor of willingness to harm the partner in a custody dispute than the “dark triad” variables which have frequently been associated to transgression. Moral disengagement captures global aspects of the tendency to ignore self-control over transgressions and seems to be a better predictor of the inclination to behave unethically against the partner.

The third hypothesis in the study stated that moral disengagement would still be a significant predictor of willingness to harm the partner in a custody dispute when controlling for the Dark Factor of Personality. Partial correlation and bifactor model results support that moral disengagement fits in a broader factor related to self-interested and detrimental behaviors, but it predicts behavior beyond that general factor. What probably makes moral disengagement different from other dark traits is the specific focus on the need to avoid moral self-sanctions and on circumventing one’s own moral principles [16], whereas beliefs associated with common core of dark traits are more focused on justifying deviation from social norms (e.g. beliefs on the adequacy of negative behavior to obtain a goal). The fit indices four our bifactorial SEM model were appropriate, except for chi-square and CFI, although sample and model size explain these discrepancies.

Our fourth hypothesis, in accordance with Hamel *et al*. [19], and Penfold [3] stated that attitudes toward unethical behavior in a child custody dispute would not be significantly different in men and women. Although men are generally commit a higher number of transgressions and antisocial behaviors in other domains, they do not lie or retaliate significantly more than women in this specific setting.

Our fifth hypothesis regarded gender differences. Men in our sample scored higher in moral disengagement than women, in line with previous research. Similar results have been found previously not only for moral disengagement [6,7], but also for related variables like those in the “dark triad” [17,30,31]. As it turns out, this result seems incongruent with our second and fourth hypothesis, because if moral disengagement predicts antisocial behavior and there are no gender differences in the target behaviors in our study, it follows that there should not be either gender differences in moral disengagement. Perhaps other variables may play a moderating role in this relationship, like empathy or negative affect [17,18] which differentially may influence antisocial behavior depending on gender. In accordance to this, moral disengagement may be a better predictor of unethical attitudes for men, at least as regards custody disputes. However, we have not tested for these possible moderators, which is a limitation in the study.

Finally, we wanted to explore whether men and women would differ in moral disengagement strategies. Although men score higher in every type of moral disengagement mechanisms, the best predictors for men and women are different. Controlling for other strategies, men tend to favor reconstrual strategies and women recipient strategies. Men tend to justify their behavior as correct and following a higher purpose (moral justification), to downplay the behavior as less negative (advantageous comparison) or to sanitize it using euphemistic labelling. In turn, the better predictor for women were recipient strategies, focused on what the victim brought upon himself to deserve retaliation or false allegations (attribution of blame) or how the recipient is somehow flawed or despicable and does not deserve the same consideration as other human beings (dehumanization).

Bersoff [32] states that men engage in unethical behaviors more frequently than women except when it is difficult to employ rationalization strategies to justify transgressions (e.g. self-serving biases are exposed or there is no motivation to use them). In our study, participants might be considered as sensitive to the situations described, since they were related to their personal experiences. However, they had nothing to gain from the hypothetical scenarios, so they were not as motivated to employ self-serving rationalization strategies as they would be in a real setting. This has two main implications. Firstly, interventions aimed at de-activating or exposing moral disengagement biases might be successful in deterring individuals from unethical behavior. On the other hand, results using hypothetical vignettes may not translate fully into real situations, since the utilitarian motivations to make unethical decisions might be weaker.

Another limitation is the correlational nature of the study that does not allow to draw causal explanations for the relationship variables. Future research must focus experimental studies and on studying settings that activate self-serving motivations. Experimental designs, case studies and real realistic situations would allow to examine the actual mechanisms involved in real transgressions. Our attempt at triggering these mechanisms this was using a sample of people who had experienced a break up with their partner and had children. Also, although hypothetical, our vignette depicted a situation of child custody dispute, where there is much to gain or lose, so it contained the features to be an adequate trigger for self-serving motivations.

The current study has also implications for practice. Being aware of the role of moral disengagement in unethical behaviors would help the professional improve their detection by using available measures. Moral disengagement measurement would allow for more precise evaluations of parents in a child custody dispute. Also, intervention programs based on moral disengagement bias detection might be devised to reduce conflict or to mediate between the partners during a break up. Acknowledging the severity of unethical behaviors, accepting responsibility for them and humanizing the recipient might be achieved by exposing the nature of moral disengagement strategies so the individuals realize that they are not acceptable rationalizations for unethical behavior.

## Supporting information

S1 Text Appendix—Vignette and questionnaires in English and Spanish. (DOCX)

## References

[1] Mazeh Y, Widrig M. The Rate of False Allegations of Partner Violence. Journal of Family Violence. 2016;31(8):1035–7. 10.1007/s10896-016-9882-3

[2] Trocme N, Bala N. False allegations of abuse and neglect when parents separate. Child Abuse & Neglect. 2005;29(12):1333–45. 10.1016/j.chiabu.2004.06.016 16293307

[3] Penfold PS. Mendacious moms or devious dads—some perplexing issues in child-custody sexual abuse allegation disputes. Canadian Journal of Psychiatry-Revue Canadienne De Psychiatrie. 1995;40(6):337–41. 7585404 10.1177/070674379504000610

[4] Smit A, Antokolskaia M, Bijleveld C. Between Scylla and Charybdis: A Literature Review of Sexual Abuse Allegations in Divorce Proceedings. Psychology. 2015;6:1373–1384. 10.4236/psych.2015.611134

[5] Barbarín MJ, Gallego G, Gómez-Villora JM, Magro V, Nadal A, de Paúl JM, et al Estudio sobre la aplicación de la ley integral contra la violencia de género por las audiencias provinciales [Study on the aplication of the integral law against gender violence in provincial courts]. Madrid: Consejo General del Poder Judicial 2016 251 p.

[6] Bandura A, Barbaranelli C, Caprara GV, Pastorelli C. Mechanisms of moral disengagement in the exercise of moral agency. Journal of personality and social psychology. 1996;71(2):364 10.1037/0022-3514.71.2.364

[7] Bandura A, Caprara GV, Barbaranelli C, Pastorelli C, Regalia C. Sociocognitive self-regulatory mechanisms governing transgressive behavior. Journal of Personality and Social Psychology. 2001;80(1):125–35. 10.1037//0022-3514.80.1.125 11195885

[8] Bandura A. Selective moral disengagement in the exercise of moral agency. Journal of Moral Education. 2002;31(2):101–19. 10.1080/0305724022014322

[9] Jakobwitz S, Egan V. The dark triad and normal personality traits. Personality and Individual Differences. 2006;40(2):331–339. 10.1016/j.paid.2005.07.006

[10] Carton H, Egan V. The dark triad and intimate partner violence. Personality and Individual Differences. 2017;105:84–8. 10.1016/j.paid.2016.09.040

[11] Detert JR, Trevino LK, Sweitzer VL. Moral disengagement in ethical decision making: A study of antecedents and outcomes. Journal of Applied Psychology. 2008;93(2):374–91. 10.1037/0021-9010.93.2.374 18361639

[12] Moore C, Detert JR, Trevino LK, Baker VL, Mayer DM. Why employees do bad things: moral disengagement and unethical organizational behavior. Personnel Psychology. 2012;65(1):1–48. 10.1111/j.1744-6570.2011.01237.x

[13] Egan V, Hughes N, Palmer EJ. Moral disengagement, the dark triad, and unethical consumer attitudes. Personality and Individual Differences. 2015;76:123–8. 10.1016/j.paid.2014.11.054

[14] Moshagen M, Hilbig BE, Zettler I. The dark core of personality. Psychological Review. 2018:125(5):656–688. 10.1037/rev0000111 29999338

[15] Jonason PK, Fletcher SA. Agentic and communal behavioral biases in the Dark Triad traits. Personality and Individual Differences. 2018:130:76–82. 10.1016/j.paid.2018.03.044

[16] Tsang JA. Moral rationalization and the integration of situational factors and psychological processes in immoral behavior. Review of General Psychology. 2002:6(1): 25–50. 10.1037/1089-2680.6.1.25

[17] Jonason PK, Kroll CH. A Multidimensional View of the Relationship Between Empathy and the Dark Triad. Journal of Individual Differences, 2015:36(3):150–156. 10.1027/1614-0001/a000166

[18] Samnani AK, Salamon SD, Singh P. Negative affect and counterproductive workplace behavior: The moderating role of moral disengagement and gender. Journal of Bussiness Ethics. 119(2): 235–244. 10.1007/s10551-013-1635-0

[19] Hamel J, Desmarais S, Nicholls T, Malley‐Morrison K, Aaronson J. Domestic violence and child custody: are family court professionals’ decisions based on erroneous beliefs? Journal of Aggression, Conflict and Peace Research. 2009;1(2):37–52. 10.1108/17596599200900011

[20] Christie R, Geis F. Studies in Machiavellianism. New York: Academic Press 1970 430 p.

[21] Corral S, Calvete E. Machiavellianism: Dimensionality of the Mach IV and its relation to self-monitoring in a Spanish sample. The Spanish Journal of Psychology. 2000;3(1):3–13. 10.1017/S1138741600005497 11761738

[22] García-Garduño JM, Cortés-Sotres JF. La medición empírica del narcisismo [The empirical measurement of narcissism]. Psicothema. 1988;10(3):725–735.

[23] Levenson MR, Kiehl KA, Fitzpatrick CM. Assessing psychopathic attributes in a noninstitutionalized population. Journal of Personality and Social Psychology. 1995;68:151–158. 7861311 10.1037//0022-3514.68.1.151

[24] Gignac GE, Watkins MW. Bifactor modeling and the estimation of model-based reliability in the WAIS-IV. Multivariate Behavioral Research, 2013:48:639–662. 10.1080/00273171.2013.804398 26741057

[25] Reise SP. The rediscovery of bifactor measurement models. Multivariate Behavioral Research, 2012:47:667–696. 10.1080/00273171.2012.715555 24049214 PMC3773879

[26] Bentler PM, Bonett DG. Significance tests and goodness of fit in the analysis of covariance structures. Psychological Bulletin, 1980:88(3):588–606. 10.1037/0033-2909.88.3.588

[27] Shi D, Lee T, Maydeu-Olivares A. Understanding the Model Size Effect on SEM Fit Indices. Educational and Psychological Measurement. 2018:1–25. 10.1177/0013164418783530 PMC6425088 30911195

[28] Steiger JH. Tests for comparing elements of a correlation matrix. Psychological Bulletin. 1980;87:245–251.

[29] Paternoster R, Brame R, Mazerolle P, Piquero A. Using the correct statistical test for the equality of regression coefficients. Criminology. 1998;36(4):859–66. 10.1111/j.1745-9125.1998.tb01268.x

[30] Muris P, Merckelbach H, Otgaar H, Meijer E. The Malevolent Side of Human Nature: A Meta-Analysis and Critical Review of the Literature on the Dark Triad (Narcissism, Machiavellianism, and Psychopathy). Perspectives on Psychological Science. 2017;12(2):183–204. 10.1177/1745691616666070 28346115

[31] Jonason PK, Davis MD. A gender role view of the Dark Triad traits. Personality and Individual Differences, 2018:125:102–105. 10.1016/j.paid.2018.01.004

[32] Bersoff DM. Why good people sometimes do bad things: Motivated reasoning and unethical behavior. Personality and Social Psychology Bulletin. 1999;25(1):28–39. 10.1177/0146167299025001003

