# Supplementary material for: Moral disengagement and willingness to behave unethically against ex-partner in a child custody dispute
Source: PLoS One. 2019 Mar 13;14(3):e0213662. doi: 10.1371/journal.pone.0213662 (PMC6415824; doi:10.1371/journal.pone.0213662)
Supplement: S1 Text — (DOCX) [file pone.0213662.s001.docx]

# **Annex 1: Child custody dispute vignettes.**

**Please try to imagine yourself in the following situation**:

The arguments with your partner are becoming worse, and you finally decide to break up. Your partner does not object to this, but he/she does have objections about your two children, aged four and nine years. You ask people who understand the judicial process for advice, and they recommend that, as above all, your partner wants to be with the children, what you should do in the trial is to issue a series of statements—no matter if they are true. This person explains that everyone does this, and you go to trial to beat the other party, and you will not regret it.

Next, please read the statements that this person proposes you to make. You rate your answers on the scale to the right, marking “1” if *you would never do that*, “2” if *you think that you would not do it*, “3” if *you might do it*, and “4” if *you would surely do it*. We remind you that this information is anonymous, so we ask for complete sincerity:

|  | 1. I would never do it | 2. I don't think I would do it | 3. I might do it | 4. I would surely do it |
| --- | --- | --- | --- | --- |
| 1. My partner regularly insulted me and despised me | 1 | 2 | 3 | 4 |
| 1. My partner physically assaulted me | 1 | 2 | 3 | 4 |
| 1. My partner treated my children badly, despised them and insulted them | 1 | 2 | 3 | 4 |
| 1. My partner hit my children and sometimes physically hurt them | 1 | 2 | 3 | 4 |
| 1. My partner earns much more money than he/she declares, and should pay a high pension for the children | 1 | 2 | 3 | 4 |
| 1. The children do not love him, they only want to be with me | 1 | 2 | 3 | 4 |
| 1. My partner never bothered with the children's food or cleanliness | 1 | 2 | 3 | 4 |
| 1. My partner knows nothing about the children's school progress, only I deal with this | 1 | 2 | 3 | 4 |
| 1. My partner's family gets on better with me than with their son/daughter | 1 | 2 | 3 | 4 |
| 1. My partner is an aggressive person with whom it is difficult to talk | 1 | 2 | 3 | 4 |
| 1. Meeting my partner was a big mistake in my life | 1 | 2 | 3 | 4 |

Two years have gone by and the relationship with the other parent is bad. There are constant problems with anything that involves the children. Now your partner has denounced you in Court, you think unfairly. Again your acquaintance advises you, and in this case, he/she proposes a number of actions. Now we ask you to answer the extent to which you would carry out such actions. Again, we remind you that this information is anonymous, so please respond sincerely:

|  | 1. I would never do it | 2. I don't think I would do it | 3. I might do it | 4. I would surely do it |
| --- | --- | --- | --- | --- |
| 1. Stop paying the children's pension that I must pay | 1 | 2 | 3 | 4 |
| 1. Prevent my partner's contact with the children when it is legally stipulated | 1 | 2 | 3 | 4 |
| 1. Instill animosity and even hatred towards the other parent in the children through my direct and indirect comments | 1 | 2 | 3 | 4 |
| 1. Convince the children that they should tell the Court technicians that the other parent treats them badly psychologically (contempt, insults) | 1 | 2 | 3 | 4 |
| 1. Convince the children that they should tell the Court technicians that the other parent treats them badly physically (hits them and attacks them) | 1 | 2 | 3 | 4 |
| 1. Attack the children physically, as I am convinced that this is the best way to attack the other parent | 1 | 2 | 3 | 4 |
| 1. Continually file complaints against the other parent (false allegations), for any reason, just so he/she will lose prestige judicially | 1 | 2 | 3 | 4 |

# **Annex 1: Escenarios de disputa por la custodia de los hijos.**

**Póngase en la siguiente situación**:

Las desavenencias con su pareja cada vez son mayores, y al final decide usted romper la convivencia. Su pareja no pone pegas al respecto, pero sí con respecto a los dos hijos que usted tiene, de cuatro y nueve años de edad. Pide consejo a personas que entienden del proceso judicial, y le recomiendan que puesto que su pareja desea ante todo estar con sus hijos, lo que debe hacer en el juicio es realizar una serie de afirmaciones que no importa que no sean ciertas, ya que le explica que es lo que hace todo el mundo y que en un juicio se va a ganar frente a la otra parte, que si no se arrepentirá.

A continuación va a leer usted las cosas que le propone esa persona que afirme. Usted debe especificar, con la escala de la derecha (señalando el “1” si nunca lo haría, el “2” si no cree que lo haría, el “3” si a lo mejor lo haría, y el “4” si seguramente lo haría. Le recordamos que esta información es anónima, por lo que le rogamos absoluta sinceridad:

|  | 1. Nunca lo haría | 2. No creo que lo hiciera | 3. A lo mejor lo haría | 4. Seguramente lo haría |
| --- | --- | --- | --- | --- |
| 1. Mi pareja me insultaba y me despreciaba de manera habitual | 1 | 2 | 3 | 4 |
| 1. Mi pareja me agredía físicamente | 1 | 2 | 3 | 4 |
| 1. Mi pareja trataba mal a mis hijos, les despreciaba y les insultaba | 1 | 2 | 3 | 4 |
| 1. Mi pareja golpeaba y a veces hacía daño físicamente a mis hijos | 1 | 2 | 3 | 4 |
| 1. Mi pareja gana mucho más dinero del que declara, y debe asumir una pensión alta para los niños | 1 | 2 | 3 | 4 |
| 1. Los niños no le quieren, y por mí se desviven | 1 | 2 | 3 | 4 |
| 1. Mi pareja nunca se ocupaba de la comida ni del aseo de los niños | 1 | 2 | 3 | 4 |
| 1. Mi pareja no sabe nada del avance escolar de los niños, solo me ocupo yo | 1 | 2 | 3 | 4 |
| 1. La familia de mi pareja se lleva mejor conmigo que con su hijo/a | 1 | 2 | 3 | 4 |
| 1. Mi pareja es una personal agresiva y con quien no se puede tratar | 1 | 2 | 3 | 4 |
| 1. El haber conocido a mi pareja ha sido un grave error en mi vida | 1 | 2 | 3 | 4 |

Han pasado dos años y las relaciones suyas con el otro progenitor son malas. Continuamente tiene problemas con todo lo que se refiere a los niños. Ahora le ha denunciado ante el juzgado, usted piensa que injustamente. De nuevo su asesor le aconseja, y en este caso le propone una serie de cuestiones. Ahora le pedimos que responda hasta qué punto usted haría tales acciones. De nuevo le recordamos que esta información es anónima, por lo que le rogamos absoluta sinceridad:

|  | 1. Nunca lo haría | 2. No creo que lo hiciera | 3. A lo mejor lo haría | 4. Seguramente lo haría |
| --- | --- | --- | --- | --- |
| 1. Dejar de pagar la pensión que me corresponde a los niños | 1 | 2 | 3 | 4 |
| 1. Evitar que mi pareja tenga contacto con los niños cuando le corresponde legalmente | 1 | 2 | 3 | 4 |
| 1. Inculcar en los niños con comentarios directos e indirectos, animadversión e incluso odio hacia el otro progenitor | 1 | 2 | 3 | 4 |
| 1. Convencer a los niños de que deben decir a los técnicos del Juzgado que el otro progenitor les trata mal psicológicamente (desprecios, insultos) | 1 | 2 | 3 | 4 |
| 1. Convencer a los niños de que deben decir a los técnicos del Juzgado que el otro progenitor les trata mal físicamente (les pega y agrede) | 1 | 2 | 3 | 4 |
| 1. Agredir físicamente a los niños, ya que estoy convencido de que esa es la mejor manera de agredir al otro progenitor | 1 | 2 | 3 | 4 |
| 1. Denunciar continuamente al otro progenitor (falsas denuncias), sin importar por qué, de forma que se le desprestigie judicialmente | 1 | 2 | 3 | 4 |
